# Wireless Stimulus-on-Device Design for Novel P300 Hybrid Brain-Computer Interface Applications

**DOI:** 10.1155/2018/2301804

**Published:** 2018-07-18

**Authors:** Chung-Hsien Kuo, Hung-Hsuan Chen, Hung-Chyun Chou, Ping-Nan Chen, Yu-Cheng Kuo

**Affiliations:** ^1^Department of Electrical Engineering, National Taiwan University of Science and Technology, Taipei, Taiwan; ^2^Center for Cyber-Physical System Innovation, National Taiwan University of Science and Technology, Taipei, Taiwan; ^3^Department of Biomedical Engineering, National Defense Medical Center, Taipei 114, Taiwan

## Abstract

Improving the independent living ability of people who have suffered spinal cord injuries (SCIs) is essential for their quality of life. Brain-computer interfaces (BCIs) provide promising solutions for people with high-level SCIs. This paper proposes a novel and practical P300-based hybrid stimulus-on-device (SoD) BCI architecture for wireless networking applications. Instead of a stimulus-on-panel architecture (SoP), the proposed SoD architecture provides an intuitive control scheme. However, because P300 recognitions rely on the synchronization between stimuli and response potentials, the variation of latency between target stimuli and elicited P300 is a concern when applying a P300-based BCI to wireless applications. In addition, the subject-dependent variation of elicited P300 affects the performance of the BCI. Thus, an adaptive model that determines an appropriate interval for P300 feature extraction was proposed in this paper. Hence, this paper employed the artificial bee colony- (ABC-) based interval type-2 fuzzy logic system (IT2FLS) to deal with the variation of latency between target stimuli and elicited P300 so that the proposed P300-based SoD approach would be feasible. Furthermore, the target and nontarget stimuli were identified in terms of a support vector machine (SVM) classifier. Experimental results showed that, from five subjects, the performance of classification and information transfer rate were improved after calibrations (86.00% and 24.2 bits/ min before calibrations; 90.25% and 27.9 bits/ min after calibrations).

## 1. Introduction

According to World Health Organization (WHO) statistics, in 2013, there were between 250,000 and 500,000 people suffering from spinal cord injuries (SCIs) [1]. Due to difficulty with mobility, an estimated 20% to 30% of people with SCIs show clinically significant signs of depression. Negative mental conditions lead to negative impacts on improvements in function and overall health. Negative attitudes and physical barriers obstruct basic mobility and result in the exclusion of people from participation in society. Improvement in the independent living ability of people suffering from SCIs is one method to overcome the disability's barriers.

Brain-computer interfaces (BCIs) are systems that interpret the brain's electrical activities to command external devices [2–6]. Thus, BCIs provide subjects with a nonmuscular method to connect with the world. Particularly for disabled people who suffer from SCIs and strokes, BCIs improve their independence in daily life. Several studies have proven that there is a great potential to develop BCIs with wide applications, such as assistive spelling systems, robotics, and rehabilitation tools. Reviewing the achievements of the past several decades, BCIs have been widely discussed. Faster, more user-friendly and more robust BCIs had been proposed by several research groups worldwide. With fast-paced technical developments, BCI studies have reached a critical point and continue to seek innovative applications.

P300 [7] is one of the conventional BCIs. P300 is an event-related potential (ERP), and the most significant feature of the potential waveform is the positive peak occurred around 300ms after a stimulus. Hence, P300 has been widely used to provide communication capabilities for healthy users, wheelchair users, and disabled people with SCIs and strokes. In addition to P300, steady state visually evoked potential (SSVEP) is another common BCI modality. SSVEP is an evoked signal that responds to visual flickering stimuli at a specific frequency. Because SSVEP is a phase-locked evoked potential, different phases of flickering stimuli are generated to obtain more stimulus targets.

Hybrid BCI is a novel architecture that integrates different BCI modalities, such as ERP, SSVEP, and motor imagery [8–11]. Particularly, auditory and tactile based P300 BCI and SSVEP BCI studies were practically proposed [12, 13]. In general, hybrid BCIs have the advantages of BCI modalities and compensate for their shortcoming. Thus, hybrid BCIs are expected to increase the accuracy and information transfer rates (ITRs). For example, P300 is a major peak and one of the most-used ERP features. The presentation of a stimulus in an oddball paradigm can elicit a positive peak in EEG. Stimuli could be visual, auditory, or somatosensory. P300 has the advantages of requiring less initial user training and being easy to observe in a simple and discriminative task. However, due to irrelative stimuli, P300-based BCI has a reported decrease in performance after long-term operation. Compared to P300, SSVEP-based BCIs [13] feature high ITRs, high signal-to-noise ratios, and relatively obvious patterns and do not require prior training procedures. However, SSVEP-based BCI requires accurate control of the subject's eye muscles and hardware that allows precisely flickering stimuli. Therefore, different paradigms of hybrid BCIs are proposed to improve single BCI modality-based systems.

A robust classifier acts an important role to recognize EEG patterns. Viewing proposed literatures, SVM [14–16], Linear Discriminant Analysis (LDA) [17–20], Bayesian Analysis [21–25], and Artificial Neural Network (ANN) [26–30] classifiers were discussed. Generally, a specific model for a user is trained before using BCI. Users are asked to follow a defined paradigm, and acquired EEG epochs are labeled particular events. Classification algorithms are trained by a labeled dataset that is called supervised learning traditionally. Type-1 fuzzy logic was proposed by Dr. Zadeh and has been discussed widely in many fields [31–35]. Type-1 fuzzy sets express the degree to which an input belongs to a fuzzy set by a crisp value. In 1975, Dr. Zadeh proposed type-2 fuzzy sets to model uncertainties. IT2FLS features interval membership functions, known as the footprint of uncertainty (FoU). FoU is expressed by two boundary functions, including a lower membership function and a higher membership function. It is robust to more uncertainties in real environments. For BCI research, because the brain's electrical signals are small and easily affected by the environment and any movement artifact, type-2 fuzzy systems show a great potential to resolve those problems. Instead of subjective fuzzy models defined by developers' knowledge, adaptive fuzzy logic systems, such as Fuzzy-Neural Network (FNN), Genetic Algorithm (GA), and swarm-based fuzzy systems, were proposed. ABC algorithm was proposed by Karaboga et al. in 2005 and was inspired by the foraging behavior of honey bee colonies [36]. It features fast convergence, less parameters, and strong robustness.

Finally, the proposed P300-based hybrid BCI with latency calibration for wireless SoD applications is elaborated in five sections in this paper, and the structure is stated as follows. In Section 1, this work specifies people with SCIs and proposes a wireless networking BCI for wireless home automation applications. In Section 2, the adopted methodologies of the proposed BCI are described. EEG processing techniques and adaptive IT2FLS for BCI calibrations are introduced. In Section 3, the experimental paradigm is specified. In Section 4, several experiments are conducted to assess the proposed hypotheses and approaches. Subjects participated in experiments to evaluate the performance of the proposed hybrid BCI.  Section 5 reviews the proposed system according to experimental results. Moreover, improved strategies and future prospections are discussed.

## 2. Methodology

This paper aims at presenting a new stimulus-on-device (SoD) design with combining the wireless sensor network (WSN) and P300-based BCI applications. Such a WSN and P300 combination forms a novel BCI application rarely seen in literatures. Such a SoD exhibits the variation of latency between target stimuli and elicited P300. Hence, this paper adopts our previous work [37] that discussed the latency problems occurred in the conventional P300 studies to deal with the subjects' P300 peak latency variation as well as the WAS transmission delay to make the SoD design practically feasible.

### 2.1. SoD Design Concept and Protocol

**Figure 1:**
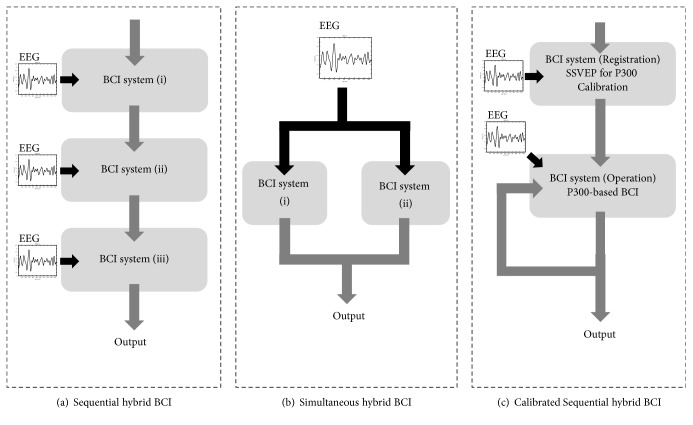
Block diagram of sequential (a), simultaneous (b), and calibrated sequential (c) (proposed) hybrid BCIs.

Considering visual-based hybrid BCI, P300 and SSVEP are two of the most discussed BCI modalities. Two main paradigms, including sequential [36, 38] and simultaneous systems [39, 40], are usually discussed. As shown in Figure 1, a sequential hybrid BCI separates a BCI system into a number of blocks. The output signals of blocks are the input signals of the next BCI system. On the other hand, a simultaneous hybrid BCI benefits individual users by obtaining a higher responded efficiency through the utilizing of an appropriate BCI modality [41]. A stimulation panel containing a number of visual stimuli is a common layout for visual-based BCIs. Stimuli responding to a defined task are arranged and presented in front of users. Users are able to select a target stimulus by gazing one of visual stimuli on a panel or a monitor. Here, this BCI layout is called SoP architecture. Instead of SoP architecture, the proposed SoD architecture embeds visual stimuli in target devices and a coordinator controller sends trigger signals to those stimulation-embedded devices through wireless communication. SoD architecture benefits the mobility of users when without using a multistimuli stimulation panel. Additionally, without the limited dimension of stimulation panel, more tasks are able to be defined by adding visual stimuli in target devices. SoD is a flexible and user-friendly BCI architecture that provides an intuitive control scheme.

**Figure 2:**
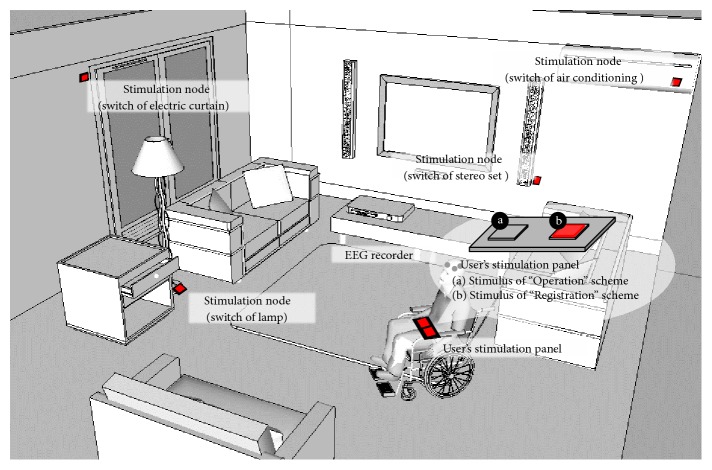
The scenario of wireless home automation based on BCI.

Based on the SoD architecture, a scenario that applies a P300-based BCI to a wireless home automation system for appliance controls, such as lighting, electric curtains, and air conditioning, is shown in Figure 2. A user is outfitted with an EEG recorder, and a coordinator controller featuring a built-in wireless communication module handles the EEG stream. A visual stimulus panel is placed in front of the user. There are two sets of LED modules that generate 16 Hz flickering visual stimuli with different phase delays. The user is able to start the BCI system by gazing at the stimulus of “Operation” and applying the P300-based BCI to the appliance controls. Then, the coordinator controller sends trigger signals to stimulation-embedded appliances. Here, each stimulation-embedded appliance is regarded as a stimulation node. The controller coordinates the latency of flashing stimuli through wireless communication and extracts epochs that respond to flashing stimuli.

**Figure 3:**
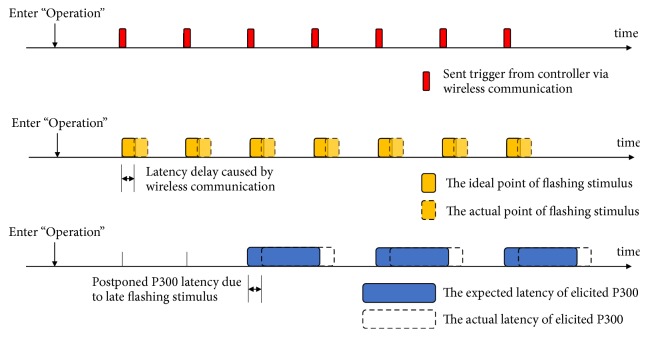
The latency of sent triggers, flashing stimuli, ideal P300 latency, and actual P300 latency.

**Figure 4:**
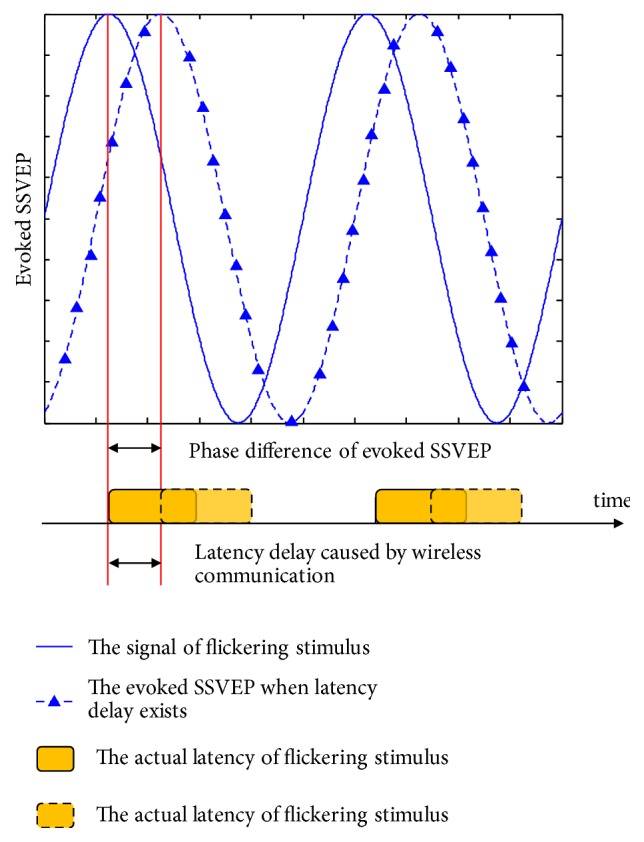
The latency of flickering stimuli and evoked SSVEP.

However, distances, transmission qualities, and hardware processing are uncertain factors that cause latency delays for this kind of SoD applications. Synchronization is influenced by any jitter between the clocks of an EEG recorder and the stimulation nodes and varies over time. The timeline of sent triggers, flashing stimuli, ideal P300 latency, and actual P300 latency are shown in Figure 3. Induced by the uncertainties of wireless communication, there are differences in the expected and actual latencies of the elicited P300 for different subjects and applied situations. Additionally, latency differences might fluctuate due to the changing quality of wireless communication. As a result, the accuracy of P300 recognition is affected while the acceptances of users decrease because of unstable performance. Therefore, a scheme of “Registration” is proposed. “Registration” allows users to access a BCI system by identifying the SSVEP. The phase lag of evoked SSVEP due to communication delays is depicted in Figure 4. This work attempts to assess how the latency of the elicited P300 correlates with the phase lag of the evoked SSVEP. A trained regression model is applied to estimate the possible latency of the elicited P300 according to phase lag analysis of the evoked SSVEP. Thus, the uncertainty of latency caused by wireless communication is resolved. Before starting “Operation”, users are asked to gaze at the stimulus of “Registration” to register their model. Users are asked to register every stimulation node item by item. A trained regression model is applied to estimate the possible latency of elicited P300 for each stimulation node. The proposed wireless P300-based BCI is calibrated when “Registration” is finished.

**Figure 5:**
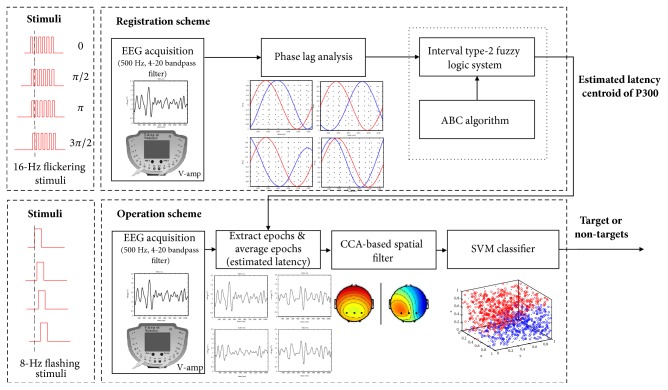
The block diagram of “Registration” and “Operation”.

The proposed wireless networking BCI includes “Registration” and “Operation” schemes. “Registration” allows users to access the BCI system by identifying the SSVEP, and “Operation” allows users to control stimulation-embedded devices via P300. A block diagram of “Registration” and “Operation” is shown in Figure 5.

### 2.2. EEG Acquisition

**Figure 6:**
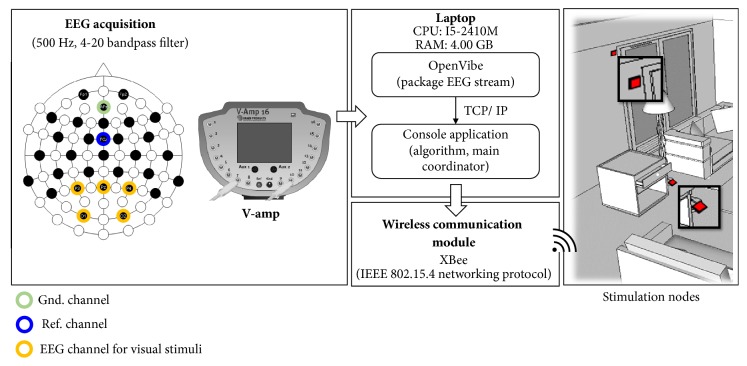
The system architecture.

This paper used the V-amp (Brain Products, German) EEG instrument to collect the subject EEG signal. The experiments were done based on the 4-20 Hz bandpass filtering frequency range. The EEG signal was acquired with 500 Hz sampling rate. Five electrodes were placed at P3, Pz, P4, O1, and O2 according to the 10/20 system. P3, Pz, P4, O1, and O2 are channels that respond to visual stimuli. The acquired EEGs were used for the phase lag analyses of SSVEP and P300 recognitions. Five electrodes were distributed in the occipital area. A reference electrode was placed at FCz, and a ground electrode was placed at AFz according to the recommended placement of the EasyCap (Standard Cap for V-amp, German). The system architecture is shown in Figure 6.

### 2.3. Signal Processing and Target/Nontarget Classification

As mentioned in the Introduction section, this paper adopts our previous work [37] to deal with the subjects' P300 peak latency variation and the WAS transmission delay. Hence, the signal processing and target/nontarget classification followed the approaches proposed in [37] as well as the experiment protocol elaborated in Section 2.1. The approaches for signal processing and target/nontarget classification are briefly described as follows.

**Figure 7:**
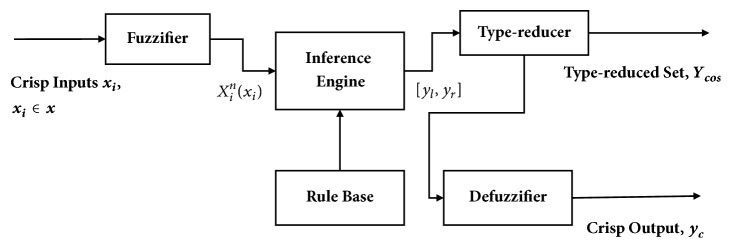
Operation of IT2FLS.

**Figure 8:**
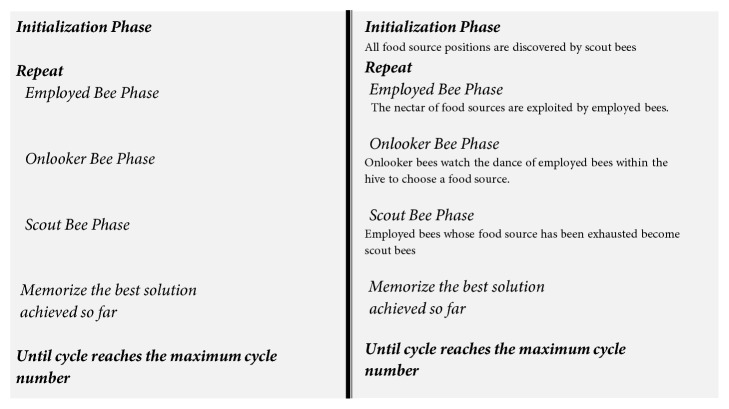
The scheme of the ABC algorithm.

**Figure 9:**
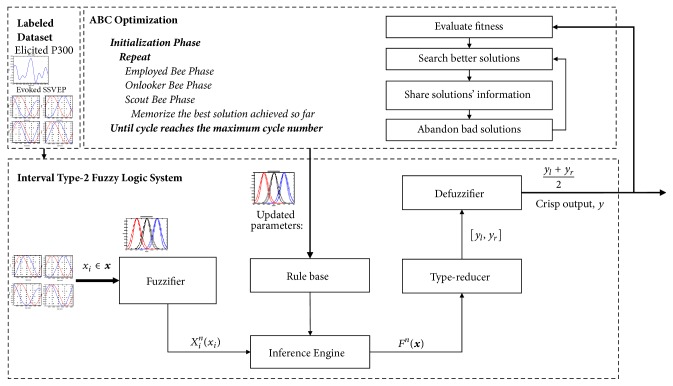
The block diagram of the proposed ABC-based adaptive IT2FLS.

(1) IT2FLS for BCI calibrations: the proposed calibration approach is to predict the latency of the elicited P300 in terms of the phase lag appeared at the 0, *π*/2, *π*, 3*π*/2 of a conventional SSVEP waveform.  Figure 7 illustrated the operation of the IT2FLS, where* x*_*i*_ is crisp inputs, *x*_*i*_^*n*^(*x*_*i*_) is the interval fuzzy set,* y*_*l*_ and* y*_*r*_ are the maximum and minimum values, respectively,* Y*_*cos*_ is generated in terms of a center-of-sets type reducer, and* y*_*c*_ is a crisp output which was obtained after the defuzzifier with respect to* y*_*l*_ and* y*_*r*_. As a consequence, the regression model based on an IT2FLS was used. The calibration was done through the analysis of phase delays. Evoked phase lags were the inputs of IT2FLS, and a calibrated latency centroid of P300 was the output for the future application bases of the SoD approach. The details can be found in [37].
(2) ABC algorithm: in this paper, the parameters of IT2FLS were evaluated in terms of the swarm-based ABC algorithm that was inspired by the honey bee colonies' foraging behaviors. The scheme of the ABC algorithm is shown in Figure 8. The details can be found in [37].
(3) Adaptive IT2FLS for BCI calibration: the block diagram of the proposed ABC-based adaptive IT2FLS is shown in Figure 9. A labeled training dataset is required to train the appropriate parameters of adaptive IT2FLS. A labeled training dataset includes a number of training trials. Each trail is a pair consisting of input values (the phase lag of evoked SSVEP) and a desired output value (the latency centroid of elicited P300). In the ABC algorithm, a fitness function describes the level of fitness of the *m*th food source, which is a D-dimensional vector consisting of the parameters of IT2FLS. The fitness function in this work is defined as (1).
  (1) fitness=1∑k=1Kgym,k,ytarg⁡et,km=1,2,…,M  k=1,2,…,K

where *y*_*m*,*k*_ is the output of the *m*th IT2FLS when the *k*th dataset is imported, *y*_*t*arg⁡*et*,*k*_ is the desired output of the *k*th dataset, and *g* is the objective function that calculates mean square errors of the difference between *y*_*m*,*k*_ and *y*_*t*arg⁡*et*,*k*_, as shown in (2). Thus, the *m*th food source is evaluated by a labeled training dataset that contains *K* datasets.

(2) gym,k,ytarg⁡et,k=ym,k−ytarg⁡et,k2m=1,2,…,M  k=1,2,…,K

### 2.4. CCA-Based Spatial Filter for Event-Related Potentials

Spatial filters are usually applied to improve the SNR of the EEG. CCA-based spatial filters have previously been used to increase the classification accuracy of evoked potentials, such as SSVEP. Spüler et al. proposed a CCA-based spatial filter for ERP [42]. Here, a CCA-based spatial filter is used to improve P300 classifications. CCA is a multivariate statistical method and can be applied to find linear transformations that maximize the correlation between the two datasets. There are two multidimensional datasets,* M* and* N*, with* q* variables. CCA is used to find two transformations,* W*_*M*_ and* W*_*N*_, to maximize the canonical correlation* ρ*, as described in

(3) U=WMTM

(4) V=WNTN

(5) ρ=covU,VvarUvarV

where* W*_*M*_ and* W*_*N*_ are two transformations that maximize the canonical correlation* ρ* between canonical variables* U* and* V*. The canonical correlations of* M* and* N* are obtained by solving eigenvalue equations, as shown in

(6) CMM−1CMNCNN−1CNMWM=ρ2WM

(7) CNN−1CMNCMM−1CMNWN=ρ2WN

where* C*_*MM*_ and* C*_*NN*_ are covariance matrices of* M* and* N*,* C*_*MN*_ and* C*_*NM*_ are covariance matrices between* M* and* N*, *ρ*^2^ is a squared canonical correlation value, and eigenvectors* W*_*M*_ and* W*_*N*_ are two transformation matrices. Here, *W*_*M*_^*T*^ is used as the spatial filter. The CCA-based spatial filter acts as a whitening filter that decorrelates signals. By maximizing the canonical correlation, the CCA finds a spatial filter that improves the SNR of the elicited potentials.

### 2.5. SVM Classifier

Given a labeled dataset {(**p**_*i*_, *q*_*i*_)}_*i*=1_^*n*^, *q*_*i*_ ∈ {1, −1}, where* p*_*i*_ are sampled EEG and *q*_*i*_ are class labels, SVM algorithm is trained by solving the following optimization problem:

(8) Minimize 12w2+C∑i=1nξi

(9) Subject  to qiwTϕpi+b−1+ξi≥0

*w* and* b* are the weight vector and the bias of hyperplane.* ϕ* maps* p*_*i*_ to a higher dimensional space and* ξ*_*i*_ are slack variables. In this work, the radial basis function (RBF) is adopted to perform nonlinear classifications for BCI.

## 3. Experimental Paradigm

Eight healthy subjects, composed of 6 males and 2 females (mean age 22 years, standard deviation three years), participated in the experiments. All subjects were students at the National Taiwan University of Science and Technology and had minimal prior experience operating visual-based BCI. All subjects had normal or corrected to normal vision.

**Figure 10:**
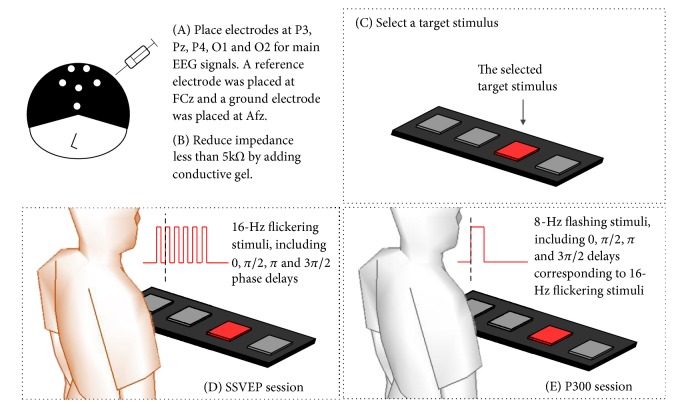
The experimental setup and paradigm.

Subjects were asked to view a stimulation panel of a 1 × 4 matrix. The stimulation panel was composed of four modules of LED arrays with the same dimensions of 3 (cm) × 3 (cm), which were placed with an interval of 8 cm between them. Each LED array presented visible red light on a black background and used moderate intensity. There was a 50 cm distance between the stimulation panel and the subjects, who were in comfortable positions. The experiments include two major components. First, a flickering stimulus was presented to observe SSVEP. A phase lag analysis was performed to realize the difference in evoked SSVEP when applying different stimulus phases. Second, a flashing stimulus was presented to observe the elicited P300. The averaging method was used to realize the difference in the elicited P300 between target and nontarget epochs. In addition, a certain delay was applied to realize the change of latency of the elicited P300. Finally, this work assessed how the latency of the elicited P300 correlated with the phase lag of SSVEP. The experimental setup is summarized in Figure 10.

### 3.1. SSVEP Session

To realize the phase lag of SSVEP when providing 0, *π*/2, *π*, and 3*π*/2 phase delays, a 16 Hz flickering stimulus was generated. To meet the scenario of the proposed BCI, the SSVEP sessions were conducted before the P300 sessions. There was a 1-minute break between the SSVEP and P300 sessions. Before starting the experiments, subjects were able to select any LED array module based on their comfort level. Each flickering stimulus continued for 5 seconds, and there was a 2-second interval between different phase stimuli.

### 3.2. P300 Session

To realize the responded latency of the elicited P300 when adding 0, *π*/2, *π*, and 3*π*/2 delays corresponding to 16 Hz flickering stimuli, an 8 Hz flashing stimulus was generated. Subjects were allowed to select an LED array module that generated the flashing stimulus. The selected LED array module was regarded as the target stimulus, and the remaining LED array modules were regarded as nontarget stimuli. To improve the SNR of the elicited P300, each stimulus flashed 30 times, and extracted epochs were further averaged. Therefore, 120 epochs in total, including 30 target and 90 nontarget epochs, were collected.

### 3.3. SVM Classifier for P300-Based BCI

An SVM classifier was used to classify extract epoch into target or nontarget stimuli. In order to evaluate the implemented BCI, an online session was simulated. P300 dataset contained an amount of 10 sessions (5 subjects with two sessions each), and there were 100 trials for each session (100 targets and 300 nontargets). The epochs of first session were used as training dataset, and the epochs of second session were used as test dataset.

## 4. Experimental Results and Discussions

### 4.1. Correlation between Elicited P300 and Evoked SSVEP

To determine how the evoked SSVEP correlated with the latency of the elicited P300, phase delays, including 0, *π*/2, *π*, and 3*π*/2, for SSVEP and flashing delays, including 0.000 (ms), 15.625 (ms), 31.250 (ms), and 46.875 (ms), for P300 were evaluated. Because a 16 Hz flickering stimulus was adopted, the reference signal peaks at 15.625 ms (*t*_ref_ = 15.625) and the phase lags were obtained according to (10), where *t*_res_ is the latency of peak of the evoked SSVEP, and* T* is the period of flickering stimuli. Additionally, the latency of the elicited P300 was recognized according to the highest response elicited potential.

(10) $$\begin{array}{c} \theta =\frac{t_{res}-t_{ref}}{T}\times 360^{{}^{\circ}} \end{array}$$

**Figure 11:**
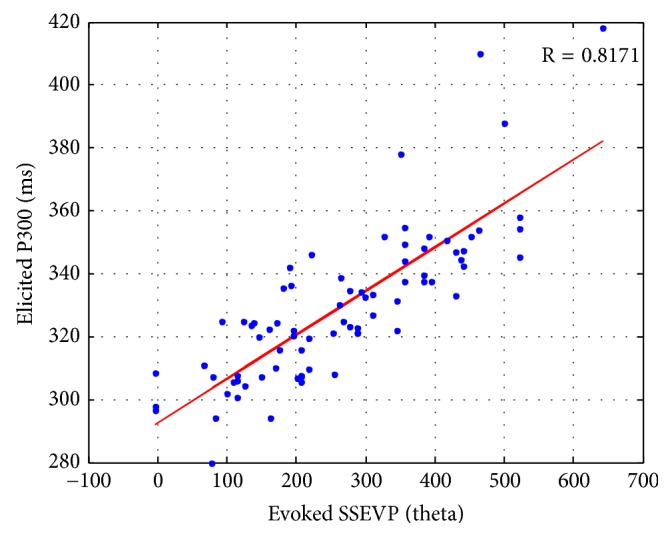
The scatter plot of elicited P300 and evoked SSVEP (8 subjects).

Eight subjects were involved in this experiment. Two datasets were collected on different days for each subject. A scatter plot showing the elicited P300 and evoked SSVEP is depicted in Figure 11. It reveals how the elicited P300 correlates with the evoked SSVEP. The correlation coefficient is computed by

(11) $$\begin{array}{c} R=\frac{cov\left(X_{P300},Y_{SSVEP}\right)}{\sigma_{X_{P300}}\sigma_{Y_{SSVEP}}} \end{array}$$

where *X*_*P*300_ is the latency of the elicited P300, *Y*_*SSVEP*_ is the phase of the evoked SSVEP, and *σ*_*X*_*P*300__ and *σ*_*Y*_*SSVEP*__ are their standard deviations. Here, the correlation coefficient is 0.8171, which indicates high correlation between the evoked SSVEP and elicited P300.

### 4.2. The Adaptive IT2FLS for BCI Calibration Based on Evoked SSVEP

**Figure 12:**
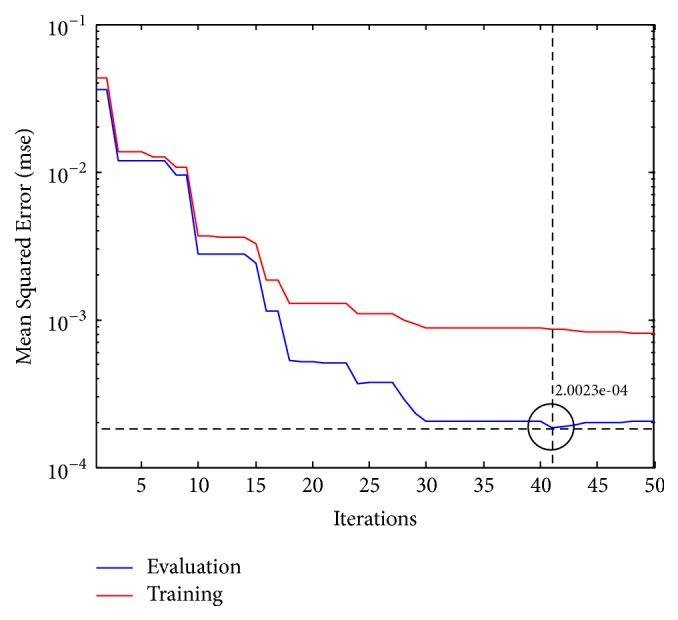
The training performance of ABC-based IT2FLS for BCI calibration.

**Figure 13:**
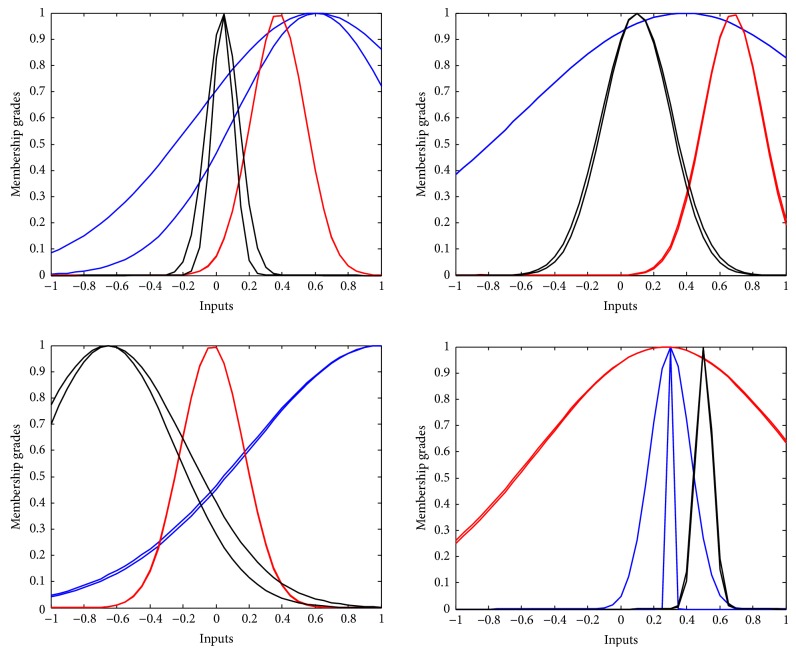
Antecedent membership functions for window calibrations.

An adaptive IT2FLS was used to calibrate the latency centroid of the extracted epochs for P300 recognitions. Phase lags were the inputs of IT2FLS, and an estimated latency centroid of P300 was the output. An adaptive IT2FLS based on ABC optimization was trained by a labeled dataset. The labeled dataset consisted of evoked phase lags (features) and P300 latency centroids (target). Collected datasets were further divided into two groups (60% for training instances; 40% for evaluation instances). The training performance is shown in Figure 12. There were three membership functions for each fuzzy set (number of fuzzy sets = 4; number of membership functions = 3), as shown in Figure 13.

### 4.3. P300 Recognition with and without Calibrations

**Figure 14:**
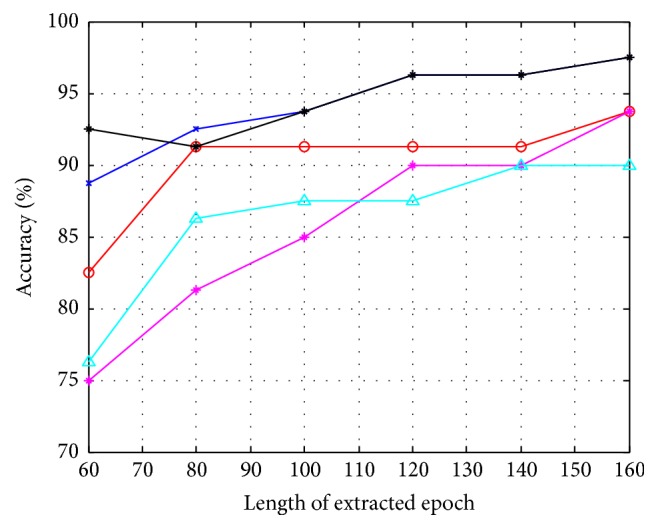
The accuracy of P300 classification depending on different lengths of extracted epochs with calibrations.

**Figure 15:**
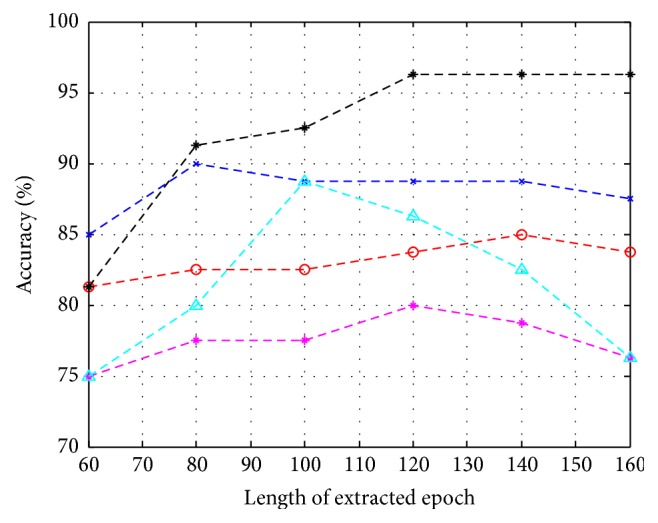
The accuracy of P300 classification depending on different lengths of extracted epochs without calibrations.

**Figure 16:**
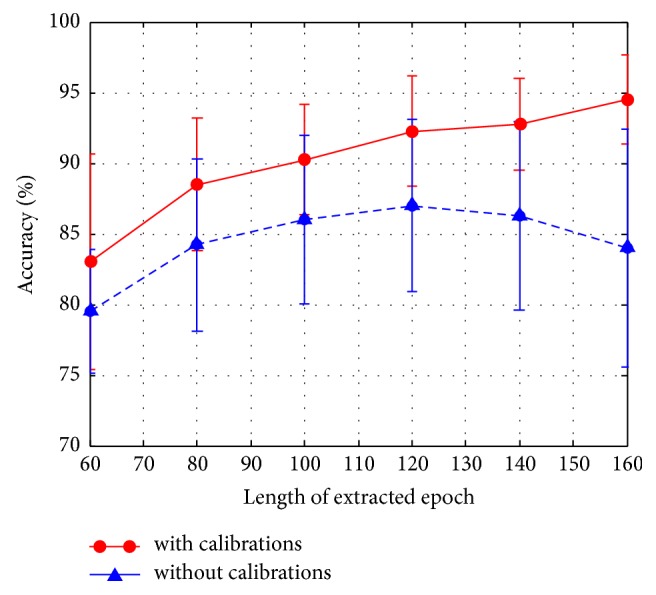
The comparison between with and without calibrations depending on different lengths of extracted epochs.

Here, a trained IT2FLS model was used to estimate the latency centroid of the elicited P300 according to the evoked SSVEP. Different lengths of extracted epochs, including 160 (ms), 140 (ms), 120 (ms), 100 (ms), 80 (ms), and 60 (ms), based on estimated centroid, were evaluated. If an estimated latency centroid was located at *t*_*c*_, an extracted epoch ranged from *t*_*c*_ − 80(ms) to *t*_*c*_ + 80(ms) when the length of the extracted epoch was 160 (ms). On the other hand, without calibrations, fixed intervals, including *t*_*s*_ + 300 to *t*_*s*_ + 460 (ms), *t*_*s*_ + 300 to *t*_*s*_ + 440 (ms), *t*_*s*_ + 300 to *t*_*s*_ + 420 (ms), *t*_*s*_ + 300 to *t*_*s*_ + 400 (ms), *t*_*s*_ + 300 to *t*_*s*_ + 380 (ms), and *t*_*s*_ + 300 to *t*_*s*_ + 360 (ms), were adopted. Here, *t*_*s*_ was the time of flashing stimulus. It was noted that 5 repetitions were conducted in this experiment, and the extracted epochs were downsampled at 62.5 Hz. The SVM classifier was employed to classify extracted epochs into target and nontarget stimuli. The accuracy rates of the SVM classifier and the ITRs with and without calibrations are shown in Figures 14–16 and Table 1. According to the experimental results, the performance of classification improves after calibration. In addition, the adopted classifier maintains satisfactory accuracy rates when decreasing the length of the extracted epoch.

## 5. Conclusion and Future Work

This work presented a wireless networking hybrid BCI to address the issue of improving the independence of individuals with SCIs. A scheme based on SoD architecture, which applies a P300-based BCI to wireless applications, was presented. However, because P300 recognitions rely on the synchronization between stimuli and elicited potential, the variation of latency between the target stimuli and elicited potential is a concern. Therefore, this work proposed “Registration” and “Operation” schemes that calibrated the latency centroid of P300 according to the evoked SSVEP and control stimulation-embedded appliances via P300. Therefore, the experiments attempted to assess how the latency of the elicited P300 correlates with the phase lag of the evoked SSVEP. Five subjects participated in the experiments, and a trained IT2FLS-based model was applied to estimate the possible latency centroid of the elicited P300 according to a phase lag analysis of the evoked SSVEP. In addition, an SVM classifier was employed to judge either target or nontarget stimuli. Based on calibrated latency centroids, different lengths of extracted epochs were discussed. Classification performances showed that the accuracy rates improve after calibration.

In the future, the uncertainty of the ERP amplitude due to different distances between users and stimulation nodes is an interesting topic. In addition, the light intensity of the operating environment is another issue in real-world applications. The modulation of appropriate stimulus intensity related to users' comfort and system performance would be another future work of this paper.

## Figures and Tables

**Table 1:** ITR (bits/min) comparisons between with and without calibrations.

	With calibrations						Without calibrations					
Subject #	1	2	3	4	5	Average	1	2	3	4	5	Average

Length of epoch	ITR						ITR					

160	31.3	35.8	35.8	31.3	27.5	32.3	22.0	25.2	34.2	16.7	16.7	22.9
140	28.7	34.2	34.2	27.5	27.5	30.4	23.0	26.3	34.2	18.3	21.1	24.6
120	28.7	34.2	34.2	27.5	25.2	29.9	22.0	26.3	34.2	19.2	24.1	25.2
100	28.7	31.3	31.3	23.0	25.2	27.9	21.1	26.3	29.9	17.5	26.3	24.2
80	28.7	29.9	28.7	20.1	24.1	26.3	21.1	27.5	28.7	17.5	19.2	22.8
60	21.1	26.3	29.9	15.8	16.7	22.0	20.1	23.0	20.1	15.8	15.8	19.0

## Data Availability

The data used to support the findings of this study are available from the corresponding author upon request.
